# The French Revolutionist, Jean Paul Marat

**Published:** 1897-05

**Authors:** J. A. Mullen

**Affiliations:** Houston, Texas


For the Texas Medical Journal.

### THE FRENCH REVOLUTIONIST, JEAN PAUL MARAT, AS A PHYSICIAN AND PHYSICIST.

BY J. A. MULLEN, M. D., HOUSTON, TEXAS.

AFTER the expurgation of Jean Paul Marat's character as a physician, and attestation of his medical success and scientific aspirations, by H. Morse Stephens in the *Pall Mall Magazine*, we feel strongly inclined, as did Caneades of old, who was accustomed to "copious doses of white hellebore, a great aperient, as a preparation to refute the dogmas of the Stoics," to imbibe the same drug and hope for a refutation of the emotional assertions of Carlyle, who called Dr. Marat "the strangest horseleech, redolent of soot and horse-drugs."

Mr. Stephens asserts that the author of "The History of the French Revolution" was prompted by prejudice and guided by ignorance in his delineation of Marat, the doctor.

The true facts of comparative chronology prove that J. P. Marat, M. D., was not a "common ill-educated horse-doctor," as Carlyle would have us believe, but show him to have been exceedingly well educated, and a linguist of no mean ability in French, Dutch, Italian, Spanish and English.

He studied medicine at the universities of Bordeaux and Toulouse, while there devoting much time to optics and electricity. He first practiced in Paris in 1762, where, in a pamphlet, he described "A Remarkable Disease of the Eye cured by Electricity, which had been considered incurable."

Residing only a short time in Amsterdam, he went to London in 1765, at the age of 22 years. While in England, his "Essay on Gleet" appeared, in 1775, and on June 30th of the same year he received an honorary degree of M. D. from the University of St. Andrews, Scotland. He also was the author of "An Inquiry of the Nature, Cause and Cure of a Singular Disease of the Eyes," which is to be found in the library of the Royal Medical and Chirurgical Society, London. In 1873, he wrote a "Philosophical Essay on Man, being an attempt to Investigate the Principles and Laws of the Reciprocal Influence of the Soul and Body," which was intended by him "as a counterblast to the popular work of Helvetius, De l' Esprit."

In 1777, June 24th, he was appointed Physician to the Body

Guard of Comte d'Artois, Louis XVI's youngest brother, at Paris.

He was especially successful in the practice of medicine, for he retired from court wealthy, just 40 years of age, to study physical science.

As a revolutionist of accepted ideas and systems, he, in the capacity of a physicist, edited, in 1779, "Dicouverte sur le Feu l' Elictricite et la Lumiere"; and in the following year published "Recherches Physiques sur l' Elictricite." His "Notions Elementaires l' Optique" and "Memoire sur l' Electricite Medicale" were issued ten years later. The latter work was "crowned" by the Academy of Ruen.

Such is the history of Jean Paul Marat, M. D., as a physician, physicist, and author. As a physicist he was the unsuccessful refutor of Newton and Franklin's theories, and if dates and facts do not lie, Carlyle's estimate of him as a physician must be radically wrong and unjust, fully deserving of Caneades' treatment. Thus history, after a lapse of over a hundred years, gives credit where credit is due, and records Marat in the Annals of Medicine in a far more appreciative sense than was intended by Carlyle. He, at least, merits this honor, no matter what fate he deserved or received as a leader of the French Revolution, or as the victim of Charlotte Corday's patriotic fury.

